# Insights on Multiple Myeloma Treatment Strategies

**DOI:** 10.1097/HS9.0000000000000163

**Published:** 2018-12-27

**Authors:** María-Victoria Mateos, Heinz Ludwig, Ali Bazarbachi, Meral Beksac, Joan Bladé, Mario Boccadoro, Michele Cavo, Michel Delforge, Meletios A. Dimopoulos, Thierry Facon, Catarina Geraldes, Hartmut Goldschmidt, Roman Hájek, Markus Hansson, Krzysztof Jamroziak, Merav Leiba, Tamás Masszi, Larisa Mendeleeva, Michael O’Dwyer, Torben Plesner, Jesús F. San-Miguel, Christian Straka, Niels W.C.J. van de Donk, Kwee Yong, Samo Zver, Philippe Moreau, Pieter Sonneveld

**Affiliations:** 1 Complejo Asistencial Universitario de Salamanca, Instituto de Investigación Biomédica de Salamanca (CAUSA/IBSAL), Salamanca, Spain; 2 Wilhelminen Cancer Research Institute, Wilhelminenspital, Vienna, Austria; 3 Department of Internal Medicine, American University of Beirut, Beirut, Lebanon; 4 Ankara University School of Medicine, Ankara, Turkey; 5 Institut d’Investigac’ons Biomedques August Pi Sunyer (IDiBAPS), Barcelona, Spain; 6 University of Torino, Azienda Ospedaliero-Universitaria Città della Salute e della Scienza di Torino, Turin, Italy; 7 Seràgnoli Institute of Hematology, Bologna University School of Medicine, Bologna, Italy; 8 University Hospital Leuven, Leuven, Belgium; 9 School of Medicine, National and Kapodistrian University of Athens, Athens, Greece; 10 Service des Maladies du Sang, Hôpital Claude Huriez, Lille, France; 11 Hospital and University Centre of Coimbra, Coimbra, Portugal; 12 Department of Internal Medicine V, University Clinic Heidelberg and National Center of Tumor Diseases, Heidelberg, Germany; 13 Department of Hematooncology, University Hospital Ostrava and Faculty of Medicine, University of Ostrava, Ostrava, Czech Republic; 14 Department of Hematology, Skåne University Hospital und Lund University, Lund, Sweden; 15 Institute of Hematology and Transfusion Medicine, Warsaw, Poland; 16 Assuta Ashdod University Hospital, Faculty of Health Science, Ben-Gurion University of the Negev, Negev, Israel; 17 3rd Department of Internal Medicine, Semmelweis University, Budapest, Hungary; 18 National Research Center for Hematology of the Ministry of Healthcare of the Russian Federation, Moscow, Russia; 19 Galway University Hospital, Galway, Ireland; 20 Vejle Hospital and University of Southern Denmark, Vejle, Denmark; 21 Clínica Universidad de Navarra, CIMA, CIBERONC, IDISNA, Pamplona, Spain; 22 Klinikum Schwabing, München, Germany; 23 Department of Hematology, VU University Medical Center, Amsterdam, The Netherlands; 24 Cancer Institute, University College London, London, United Kingdom; 25 University Clinical Center Ljubljana and Medical Faculty, Ljubljana, Slovenia; 26 University of Nantes, Nantes, France; 27 Department of Hematology, Erasmus MC Cancer Institute, Rotterdam, The Netherlands

## Abstract

The introduction of new agents and management strategies over the past decade has resulted in a major step change in treatment outcomes with deepening responses and increased survival for patients with multiple myeloma. In daily clinical practice, healthcare professionals are now faced with challenges including, optimal treatment sequencing and changing treatment goals. In light of this, a group of experts met to discuss diagnostic and treatment guidelines, examine current clinical practice, and consider how new clinical trial data may be integrated into the management of multiple myeloma in the future.

## Introduction

The increasing number of therapeutic options for patients with multiple myeloma (MM), both in the newly diagnosed and relapsed/refractory settings, has led to improved outcomes including prolonged survival.^1–3^ The increase in available therapies and the development of others has led to an exponential growth in clinical trial data and a rapidly changing regulatory environment.

A meeting of experts was convened to discuss diagnostic and treatment guidelines, survey current clinical practice and consider how new data may influence the management of MM across the course of the disease. This review summarizes these discussions and the supporting evidence relevant for clinical practice.

## Diagnosis and risk assessment

As might be expected, bone marrow assessment (biopsy or aspirate) remains key in the establishment of a diagnosis of MM in clinical practice, as reflected in the updated International Myeloma Working Group (IMWG) diagnostic criteria,^4^ and is performed routinely. The creatinine clearance test or estimated glomerular filtration rate (calculated using either the Modification of Diet in Renal Disease [MDRD] study or Chronic Kidney Disease Epidemiology Collaboration [CKD-EPI] equations^5,6^) are also performed at diagnosis to assess renal function. In addition, the levels of monoclonal (M) proteins and free light chains (FLC) in blood are assessed routinely to confirm active disease, although the evaluation of M-protein in urine is less widespread across clinical practice. Whole body low dose computed tomography (CT) or magnetic resonance imaging (MRI) are now recommended by the European Myeloma Network (EMN) and the IMWG as standard for the detection of lytic or focal lesions, as these techniques are more sensitive for detecting bone disease than conventional skeletal survey using X-ray.^4,7,8^ Whole body low dose CT is a valid technique for the evaluation of bone lesions and whole body MRI is effective for the evaluation of bone marrow. MRI of the spine and pelvis are particularly valuable for detection of focal lesions and may be used if whole body imaging is not available.^7^ In addition, positron emission tomography (PET)-CT may be useful in determining treatment response and disease progression.^7,9^ However, some of the newer imaging techniques are not available in all countries or outside larger Centres of Excellence, and in this case conventional radiological techniques can be used.

According to the IMWG, the correct diagnosis of MM requires a number of procedures.^4^ A bone marrow biopsy (or aspirate) is needed to determine a percentage of ≥10% clonal bone marrow plasma cells (clonality requires demonstration of κ/λ-light-chain restriction on flow cytometry, immunohistochemistry, or immunofluorescence) and the presence of bony or extramedullary plasmacytoma. The identification of at least one myeloma-defining event is also required to confirm diagnosis. Hypercalcemia is determined using serum calcium concentration, renal insufficiency by measuring creatinine clearance (using the MDRD or CKD-EPI equations) or serum creatinine concentration, anemia by measuring hemoglobin value, and the presence of osteolytic lesions using skeletal radiography, CT, or PET-CT. Finally, a set of biomarkers of malignancy have been identified as additional “myeloma defining events”: bone marrow biopsy is used to confirm ≥60% clonal bone marrow plasma cells, MRI to identify the presence of one or more focal lesions, and the free light chain (FLC) assay to determine an abnormal FLC κ/λ ratio of ≥100.

The validated Revised International Staging System (R-ISS) is widely used as a staging system at diagnosis.^10^ Specifically, cytogenetic testing typically performed by fluorescence in situ hybridization on CD138-selected cells, is used increasingly in clinical practice because of the impact of cytogenetic risk on treatment choice.^10,11^ Although potentially useful in helping clinicians make treatment decisions, currently available frailty scores (e.g., the IMWG score and the revised Myeloma Comorbidity Index)^12,13^ are of limited value in everyday clinical practice due to their complexity and time-consuming nature and as a result, are not used systematically in the majority of countries. Given the ageing population, standardized tools are needed to aid objective and accurate assessment of frailty, the subsequent treatment choice (including autologous stem cell transplantation [ASCT] eligibility) and dose stratification. In the future, patients may be stratified according to their fitness for treatment (including transplantation), rather than as transplant/nontransplant candidates by age.

*Conclusion*: Both the updated IMWG diagnostic criteria and the R-ISS have been widely adopted for use across Europe. The broader availability of newer imaging techniques and cytogenetic testing, and the development of standardized tools for the stratification of patients according to fitness, may further improve diagnosis and risk assessment in MM.

## Smouldering multiple myeloma

According to a survey among experts during our meeting, approximately 15% of patients with MM seen in clinical practice present with smouldering multiple myeloma (SMM). This estimate is similar to a prevalence of 13.7% reported in a large US study among patients with MM diagnosed between 2003 and 2011 and 14.4% in a Swedish population-based study.^14,15^ However, these studies include patients diagnosed before the publication of the updated criteria for the diagnosis of MM.^4^

The introduction of the new diagnostic criteria, including new biomarkers (plasma cell bone marrow infiltration ≥60%, serum FLC ratio ≥100, and more than one focal lesion visible on MRI [5 mm or greater]), has the potential to allow earlier treatment initiation and subsequently improve prognosis. However, this must be confirmed in prospective randomized trials.^4^ Our survey revealed that although these new biomarkers have some pitfalls, with the use of serum FLC ratio and the 5 mm cut-off for focal lesions remaining controversial, they may prove valuable in including more patients in clinical trials and to avoid the development of serious events such as renal insufficiency or bone lesions. It should be noted that the definition of monoclonal gammopathy of unknown significance (MGUS) has not been changed.^4^

The classification of patients with SMM according to their risk of progression to MM may prove useful in the management of this disease, and in improving patient access to some clinical trials. The validated Mayo Clinic and Spanish PETHEMA risk models can be applied in clinical practice,^16,17^ however the dynamic evolution of the M-component, the strongest indicator for the risk of progression, is widely used.^18^

Current guidelines do not recommend immediate treatment for patients with high-risk SMM and call for further clinical studies.^19^ There are many ongoing trials exploring the benefit of early intervention with 2 different goals: to delay clinical progression; or to achieve cure.

A phase 3 study (N = 119) demonstrated that lenalidomide-dexamethasone (Rd) prolongs time to progression of SMM to active disease, compared with observation (median time to progression not reached vs 23 months at median follow up of 75 months; hazard ratio [HR] 0.24; 95% CI: 0.14–0.41; *P* < 0.0001).^20,21^ Median overall survival (OS) was not reached in either group (HR: 0.43 [95% CI: 0.21–0.92], *P* = 0.024). It should be noted that this was a small trial without modern bone imaging at baseline, thus some patients may have already had active MM (based on novel criteria) at the time of enrolment. In a phase 2 study (median follow-up, 15.9 months), all patients with high-risk SMM (n = 12) treated with carfilzomib, lenalidomide, and dexamethasone (KRd) achieved at least a very good partial response (VGPR).^22^

Another small phase 2 study (N = 39) showed that elotuzumab, lenalidomide, and dexamethasone (EloRd) treatment in patients with high-risk SMM resulted in an overall response rate (ORR) of 71%.^23^ Two other phase 2 studies have investigated the use of monoclonal antibodies (mAbs) in the treatment of SMM. Daratumumab monotherapy was shown to have activity in intermediate- and high-risk SMM, especially among patients who received a “long” dosing regimen (N = 41; cycle 1, once-weekly; cycles 2–3, every 2 weeks; cycles 4–7, every 4 weeks; cycle 8–20, every 8 weeks), with an ORR of 56% at a median follow-up of 9.6 months.^24^ The anti-interleukin-6 mAb, siltuximab, also demonstrated single-agent activity in patients with intermediate- or ultra-high-risk SMM (N = 85), with a 1-year progression-free survival (PFS) rate of 84.5% versus 74.4% for patients receiving placebo—although the results should be interpreted with caution due to the higher proportion of ultra-high-risk patients in the placebo group.^25^

These approaches are focused on the delay of progression to MM, but the goal of 2 ongoing clinical trials is to cure asymptomatic MM patients based on strategies similar to that used in patients with active MM, including induction, transplant, consolidation and maintenance, with second-generation drugs and daratumumab.^26,27^ Early results from the GEM-CESAR study showed that among the 43 patients who had received KRd induction, there was an ORR of 98%, with 83% of patients achieving at least a VGPR.^28^

*Conclusion*: SMM represents a high proportion of patients presenting with myeloma. Although current European treatment guidelines do not recommend the immediate treatment of SMM, the updated IMWG diagnostic criteria allow patients with ultra-high-risk SMM to be redefined as having MM and therefore to be candidates for treatment. Moreover, current data suggest that early intervention in high-risk SMM translate into delayed progression to active myeloma and in at least one study, overall survival benefit. To date, clinical studies of SMM treatment have considered 2 alternative strategies: delay progression; or cure. Further studies are required to elucidate the most appropriate option.

## Front-line transplant setting

### Induction

Treatment goals for individual patients may change as their disease progresses. Among “fit” patients with newly diagnosed MM, OS is a very important goal, as are depth and duration of response. Similarly, it is important that treatment can prolong PFS. In contrast, there is a more mixed picture with regards to the importance for quality of life, tolerability, treatment duration, convenience, and patient preference at this stage of disease.

According to our survey of current practice among experts, induction is generally comprised of bortezomib-based triplet regimens, with bortezomib, thalidomide and dexamethasone (VTD) and bortezomib, cyclophosphamide and dexamethasone (VCD) the most frequently used combinations across Europe, and bortezomib, doxorubicin and dexamethasone (PAD) a less common option. Nevertheless, the triplet bortezomib, lenalidomide, and dexamethasone (VRD) is emerging as a preferable, but not yet approved, option in several European countries. These findings support the current European Society for Medical Oncology (ESMO) guidelines, which recommend induction therapy with a triplet regimen (VTD, VCD, PAD, or VRD) followed by high-dose therapy with ASCT, as standard treatment for fit patients (<65 years of age or <70 years of age in good clinical condition).^19^

Induction with VRD was shown to be an effective regimen in the phase 3 GEM2012MENOS65 study (N = 458), with an ORR of 85% (39% complete response [CR], 29% VGPR, and 17% partial response [PR]).^29^ The phase 2 IFM2008 study also showed good response rates with VRD induction (≥VGPR 58%), which were further improved after ASCT (≥VGPR 70%) and VRD consolidation (≥VGPR 87%).^30^ Other combinations are being investigated as alternatives to VRD.

Several studies are investigating novel proteasome inhibitor (PI)-based triplet combinations as induction therapy in transplant-eligible patients. A large, Italian phase 2 study (N = 281) showed higher rates of patients achieving at least VGPR with KRD compared with carfilzomib, cyclophosphamide, and dexamethasone (KCD) (74% vs 61%; *P* = 0.05). KRD was associated with more hematological grade 3–4 adverse events, but no significantly increased cardiovascular toxicity, compared with KCD.^31^ Similar response rates were observed in the EMN phase 2 study of carfilzomib, thalidomide, and dexamethasone (KTD) induction (N = 137), with 65% of patients achieving at least a VGPR and 18% at least a CR.^32^ In the UK-based Myeloma XI phase 3 study (N = 2568), the quadruplet carfilzomib, cyclophosphamide, lenalidomide, and dexamethasone (KCRD) resulted in deeper overall responses, with a higher percentage of patients achieving at least VGPR (78.7%), compared with the triplets CTD (cyclophosphamide, thalidomide, and dexamethasone; 52.9%) and CRD (60.6%).^33^ Ixazomib, lenalidomide, and dexamethasone (IRd) induction has also been investigated in the IFM phase 2 study (N = 42), with a 12% CR rate achieved after 3 cycles.^34^ An ongoing phase 3 study is evaluating VTD plus daratumumab versus VTD as induction and consolidation.^35^

*Conclusion*: Key treatment goals in fit, transplant-eligible patients are extended survival and depth and duration of response. Triplets based on a PI plus an immunomodulatory drugs (IMiD) are the preferable option. Bortezomib-based triplet therapies are the current standard of care. Positive data are emerging for other PI-based induction therapies, particularly those containing ixazomib or carfilzomib in combination with lenalidomide or cyclophosphamide and dexamethasone.

### ASCT

As previously mentioned, the ESMO guidelines recommend triplet regimen induction followed by high-dose therapy with ASCT as standard treatment for fit patients.^19^ Recent studies support this. In the ongoing EMN02/HO95 MM phase 3 study (N = 1192), high-dose intensification therapy followed by ASCT demonstrated significantly prolonged PFS compared with standard intensification therapy including 4 cycles of VMP (median PFS, not reached vs 44 months; HR, 0.76; 95% CI: 0.64–0.91; *P* = 0.002), at a median follow-up of 38 months.^36^ However, no difference in OS has been observed in the overall study population so far after a very short follow-up period.^36^ ORR was also higher with ASCT compared with VMP (84% vs 75%; *P* < 0.001).^36^ Similarly, the IFM 2009 study (N = 700) demonstrated that VRD plus ASCT is superior to continuous treatment with VRD 8 cycles alone, with PFS of 50 months versus 36 months, respectively (HR, 0.65; *P* < 0.001), at a median follow-up of 44 months; median OS was not reached.^37^ Furthermore, a greater percentage of patients in the ASCT group achieved CR compared with the VRD group (59% vs 48%, *P* = 0.13), and a greater percentage showed MRD negativity (sensitivity 10^−4^; 79% vs 65%, *P* < 0.001).^37^

Data from the EMN02/HO95 MM study also showed that upfront double ASCT was superior to single ASCT particularly in patients with high-risk cytogenetic status, prolonging PFS (HR, 0.42; 95% CI: 0.21–0.84; *P* = 0.014) and OS (HR, 0.52; 95% CI: 0.28–0.98; *P* = 0.042) in this subgroup.^38^ Moreover, double ASCT overcame the poor prognosis associated with high-risk compared with standard-risk cytogenetics.^38^

*Conclusion*: ASCT remains integral to the management of newly diagnosed MM. ASCT increases response rate, depth of response, MRD negativity, and PFS, when used following induction with a novel triplet regimen. So far no OS benefit has been observed, possibly due to limited follow-up. Tandem ASCT may be beneficial in patients with high-risk cytogenetic abnormalities.

### Consolidation

Two recent studies have evaluated VRD consolidation following ASCT. The EMN02/HO95 MM study interim analysis showed that VRD consolidation therapy is superior to no consolidation, with the exception of patients with high-risk cytogenetic abnormalities.^39^ In contrast, the US STaMINA study found no difference between patients who did or did not receive VRD consolidation after ASCT.^40^ Although these findings appear contradictory, it is important to take into account the differences in study designs and populations, and the relatively short follow-up.

A number of other regimens have been investigated. Bortezomib consolidation (16–20 doses) after ASCT has demonstrated improvements on PFS but no differences on OS to date.^41–43^ In addition, VTD consolidation was shown to be superior to TD consolidation in a phase 3 study by the GIMEMA group.^44^

*Conclusion*: Current ESMO treatment guidelines conclude that there is not sufficient evidence in favour of consolidation therapy, with longer follow-up needed.^19^

### Maintenance

According to our expert survey, maintenance treatment is increasingly becoming standard clinical practice, with lenalidomide or bortezomib the chosen regimens.

A meta-analysis of lenalidomide maintenance using patient-level data (N = 1209) from 3 phase 3 studies demonstrated a significant 2.5-year benefit in terms of OS.^45^ At a median follow-up of 80 months, median OS had not been reached for patients who received lenalidomide maintenance versus 86 months for those who did not (HR, 0.74; 95% CI: 0.62–0.89; *P* = 0.001). The benefit was not as substantial in patients with high-risk cytogenetics.^45^ However, results from the Myeloma XI phase 3 study (n = 1970) showed that at a median follow-up of 28.7 months, lenalidomide maintenance reduced the risk of progression in both transplant eligible (median PFS, 60.3 vs 30.1 months; HR, 0.47; 95% CI: 0.39–0.57; *P* < 0.0001) and noneligible patients (25.7 months vs 11.0 months; HR, 0.44; 95% CI: 0.37–0.53; *P* < 0.0001), but also in patients with high- (HR, 0.30) and ultra-high-risk cytogenetics (HR, 0.31), compared with observation.^46^

The phase 3 HOVON-65/GMMG-HD4 study demonstrated improved response and survival among patients receiving bortezomib-based induction (PAD) followed by bortezomib maintenance compared with those receiving vincristine, doxorubicin, and dexamethasone (VAD) induction and thalidomide maintenance.^47^ Bortezomib was also better tolerated than thalidomide. Long-term follow-up (median 96 months) showed that the benefits on PFS with the bortezomib regimen were maintained (median PFS 34 months [PAD] vs 28 months [VAD]; HR, 0.76; *P* < 0.001).^48^ Another phase 3 study, GEM05MENOS65 (N = 390), demonstrated that maintenance with bortezomib plus thalidomide significantly increased PFS compared with thalidomide or alfa-2b interferon at a median follow-up of almost 5 years (median PFS, 50.6 vs 40.3 vs 32.5 months; *P* = 0.03).^49^ However, no significant differences were seen for OS.^49^

Lenalidomide is now the standard of care and the only drug approved by the European Medicines Agency (EMA) as monotherapy for the maintenance treatment of adult patients with newly diagnosed MM who have undergone ASCT.^50^ ESMO guidelines recommend lenalidomide maintenance following ASCT in transplant-eligible patients.^19^ The optimal duration of maintenance is still an issue for debate.

*Conclusion*: Lenalidomide maintenance following ASCT is considered standard of care and as such is recommended by ESMO and approved by the EMA. The benefits of lenalidomide maintenance among those with high-risk cytogenetics are not clear and these patients may require a different approach.

## Front-line nontransplant setting

Although OS and long PFS are still important treatment goals among “frail” patients with newly diagnosed MM, they may be less important than in “fit” patients. Quality of life, tolerability and duration of treatment however, are of increased importance when considering treatment options in the nontransplant setting. Depth and duration of response are likely to become more important in the future, due to the increasing use of regimens associated with high response rates in this patient population.

Based on the expert survey, bortezomib plus melphalan and prednisone (VMP)/modified VMP are the most widely used regimens in the nontransplant setting, followed by VRd and VCD. The use of lenalidomide plus low-dose dexamethasone (Rd) is expected to increase in the future.

The GEM2010 phase 2 study evaluated sequential or alternating administration of VMP and Rd among newly diagnosed, elderly patients (N = 233).^51^ The results showed equivalent efficacy between the sequential and alternating arms, in terms of 18-month PFS (74% vs 80%, respectively; *P* = 0.21).^51^ Notably, achieving MRD negativity versus remaining MRD positive was not only associated with better outcomes, but also appeared to abrogate the adverse prognosis associated with high-risk cytogenetics.^52^

Maintenance with bortezomib plus thalidomide (VT) or prednisone (VP) was evaluated in 178 elderly patients with newly diagnosed MM.^53^ Both regimens had acceptable safety profiles and led to increased CR rates and prolonged PFS and OS; VT was associated with longer PFS and OS than VP. The use of maintenance following completion of initial therapy is currently being evaluated with ixazomib,^54^ while the use of lenalidomide maintenance following melphalan, prednisone and lenalidomide (MPR-R) in the MM-015 study resulted in longer PFS compared with MPR or MP alone.^55^

A number of ongoing trials are investigating lenalidomide-based combinations including, elotuzumab plus Rd (EloRd),^56^ IRd,^57^ and daratumumab plus Rd (DaraRd),^58^ compared with Rd in transplant-ineligible patients.

The oral combination of ixazomib, thalidomide, and dexamethasone is being studied in transplant-ineligible patients (N = 120) in the HOVON-126/NMSG 21.13 study.^59^ Early results have shown good response (ORR 81%), which was observed irrespective of cytogenetics or frailty, however longer follow up is required to assess PFS.^59^

Recently, the interim analysis of a phase 3 study (N = 706) comparing daratumumab plus VMP (D-VMP) versus VMP alone in transplant-ineligible patients with newly diagnosed MM showed that at a median follow-up of 16.5 months, D-VMP led to significantly longer PFS than VMP alone (HR, 0.50; 95% CI: 0.38–0.65; *P* < 0.001).^60^ OS data are not yet mature. The ORR (90.9% vs 73.9%; *P* < 0.001), and rates of VGPR or greater (71.1% vs 49.7%, *P* < 0.001) and CR or greater (42.6% vs 24.4%; *P* < 0.001) were also significantly higher with D-VMP than VMP.

The ESMO 2017 guidelines recommend VMP/modified VMP, Rd or VRd as the first option for newly diagnosed patients who are ineligible for transplant, with melphalan, prednisone, thalidomide (MPT) or VCD as second options.^19^ The experts at our meeting were in agreement with this guidance. For transplant-ineligible patients with high risk cytogenetics, Rd does not appear to improve survival outcomes.^11,61^

*Conclusion*: Treatment goals for frail, transplant-ineligible patients are shifted towards patient-centered outcomes including QoL and tolerability. In agreement with ESMO guidelines, VMP/modified VMP, VRd, and VCD are commonly used regimens in clinical practice in the nontransplant setting, with the use of Rd increasing. Studies of IMiD-based combinations and the addition of mAbs are ongoing. Furthermore, D-VMP may be approved soon.

## Assessment at relapse

Our survey revealed that frailty assessments, and cytogenetic analyses are not used routinely at relapse. However, cytogenetics and fitness are important for treatment selection.

Evaluation of M-protein in blood remains the key assessment at relapse. In addition, both creatinine clearance and serum FLC are assessed routinely at relapse. As previously mentioned, the use of a skeletal X-ray survey is no longer standard practice^4,7,8^; other imaging techniques are favored, for example, PET-CT for patients in whom there is a clinical suspicion of extramedullary progression or those with high serum lactate dehydrogenase.^62,63^ Although bone marrow evaluations are not performed as standard at relapse in clinical practice, they may be of value in certain patients, for example, those with longstanding disease who may experience “light chain escape” or even completely cease production of M-protein. Bone marrow sampling also affords the opportunity to undertake cytogenetic assessment at relapse, which may become increasingly important for selection of relapse therapy.

## Relapsed or refractory setting

In keeping with current practice, benefits on survival, along with the depth and duration of response are considered very important treatment goals in patients with relapsed disease. As therapy is usually continued until the next progression, treatment duration is not a relevant question in this setting as it is for frontline therapy. Neither quality of life nor tolerability are considered as important as treatment response and duration at first relapse, reflecting the fact that physicians and patients are willing to compromise and accept some toxicities in order to achieve long-term benefits.

Although there are no formal definitions for early and later relapses, in practice a relapse occurring within 1 year of the last line of therapy is considered early, whereas those occurring >1 year after the last line are considered later relapses.

### Early relapses

**Figure 1:**
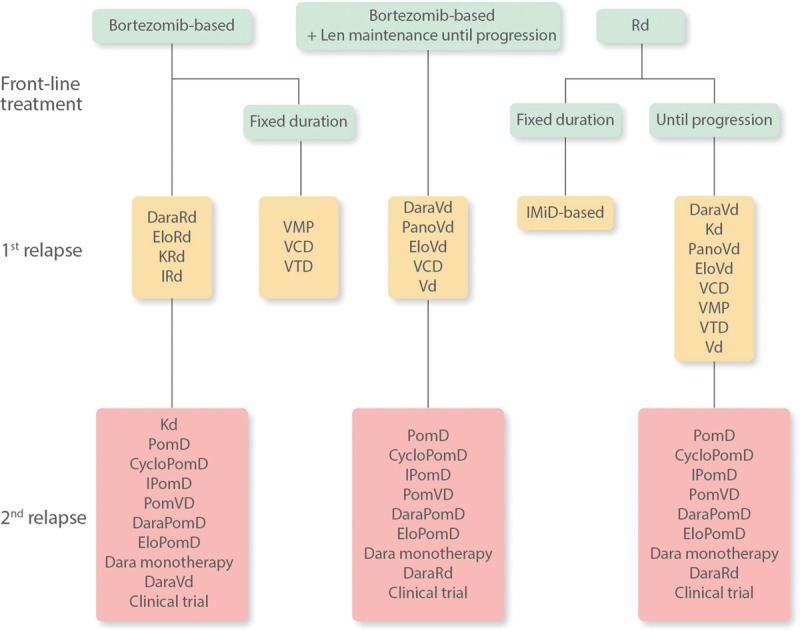
**Current treatment algorithm for patients with multiple myeloma at first and second relapse, as judged by expert panel**. CycloPomD = cyclophosphamide, pomalidomide, and dexamethasone, Dara = daratumumab, DaraPomD = daratumumab, pomalidomide, and dexamethasone, DaraRd = daratumumab, lenalidomide, and dexamethasone, DaraVd = daratumumab, bortezomib, and dexamethasone, EloPomD = elotuzumab, pomalidomide, and dexamethasone, Elo-Rd = elotuzumab, lenalidomide, and dexamethasone, Elo-Vd = elotuzumab, bortezomib, and dexamethasone, IPomD = ixazomib, pomalidomide, and dexamethasone, IRd = ixazomib, lenalidomide, and dexamethasone, Kd = carfilzomib and dexamethasone, KRd = carfilzomib, lenalidomide, and dexamethasone, Len = lenalidomide, Pano-Vd = panobinostat, bortezomib, and dexamethasone, PomD = pomalidomide and dexamethasone, PomVD = pomalidomide, bortezomib, and dexamethasone, Rd = lenalidomide and dexamethasone, VCD = bortezomib, cyclophosphamide, and dexamethasone, Vd = bortezomib and dexamethasone, VMP = bortezomib, melphalan, prednisolone, VTD = bortezomib, thalidomide, and dexamethasone.

Figure 1 presents a simplified treatment algorithm for patients at first relapse, as proposed during our meeting. In considering the various treatment options, it should be noted that there have been no head-to-head comparisons between the newer agents.

Current ESMO guidelines recommend Rd or an Rd-based triplet (e.g., DaraRd, KRd, IRd, or EloRd) for patients at first relapse after frontline bortezomib treatment.^19^ This is supported by a number of recent phase 3 studies.

Updated analyses from the POLLUX study (N = 569) have shown that DaraRd significantly extended PFS compared with Rd (at a median follow-up of 25.4 months, median PFS was not reached for DaraRd vs 17.5 months for Rd; HR, 0.41; 95% CI, 0.31–0.53; *P* < 0.0001).^64^ The ASPIRE study (N = 792) demonstrated that KRd significantly prolonged PFS compared with Rd (26.3 months vs 17.6 months; HR, 0.69; 95% CI, 0.57–0.783; *P* = 0.0001).^65^ An OS benefit for KRd over Rd was recently reported (median 48.3 months for KRd vs 40.4 months for Rd; HR, 0.79; 95% CI: 0.67–0.95; *P* = 0.0045; median follow-up of approximately 67 months).^66^ The TOURMALINE-MM1 study (N = 722) showed that IRd significantly reduced the risk of disease progression or death by 26% compared with Rd (median PFS 20.6 months vs 14.7 months; HR, 0.74; 95% CI, 0.59–0.4; *P* = 0.01) at a median follow up of 14.7 months.^67^ Updated data from the ELOQUENT-2 study (N = 646) indicated that EloRd reduced risk of disease progression or death by 27% versus Rd at median follow-up of 32.4 months (median PFS 19.4 months vs 14.9 months; HR, 0.73; 95% CI, 0.60–0.89; *P* = 0.0014).^68^

Rd-based combinations should be selected based on efficacy as well as toxicity profile and comorbidities. Patient preferences and oral versus intravenous administration can be also taken into account.

For patients who relapse after receiving an IMiD-based therapy in the frontline setting, the ESMO guidelines recommend bortezomib-based regimens such as DaraVd, Panobinostat-Vd, EloVd, or bortezomib plus cyclophosphamide and dexamethasone (VCD), or proteasome inhibitor-based doublets (Kd, Vd).^19^

Updated results from the phase 3 CASTOR study confirmed that DaraVd significantly prolonged PFS compared with Vd (median 16.7 months vs 7.1 months at a median follow-up of 19.4 months; HR, 0.31; 95% CI, 0.24–0.39; *P* < 0.0001).^69^ The PANORAMA 1 phase 3 study (N = 768) showed that panobinostat-Vd (PanoVd) significantly prolonged PFS compared with Vd (median 11.99 months vs 8.08 months; HR, 0.63, 95% CI, 0.52–0.76; *P* < 0.0001; median follow-up of 6.47 months vs 5.59 months),^70^ with greatest benefit seen for patients who received at least 2 prior regimens including bortezomib and an IMiD (12.5 months vs 4.7 months; HR, 0.47).^71^ In the final OS analysis, no difference in median OS was observed between the PanoVd (40.3 months) and Vd groups (35.8 months; HR, 0.94, 95% CI, 0.78–1.14; *P* = 0.54).^72^ A phase 2 study (N = 152) showed that median PFS was longer with EloVd (9.7 months) compared with Vd (6.9 months; HR, 0.72; 70% CI, 0.59–0.88; *P* = 0.09) at a median follow up of 15.9 months for the EloVd group and 11.7 months for the Vd group.^73^ A pre-specified interim analysis for the ENDEAVOR phase 3 study (N = 929) has shown that Kd was superior to Vd with a median PFS of 18.7 months versus 9.4 months (HR, 0.53; 95% CI, 0.44–0.65; *P* < 0.0001), at a median follow-up of 11.9 and 11.1 months, respectively.^74^ A subsequent interim analysis demonstrated that Kd prolonged median OS (47.6 months) compared with Vd (40.0 months; HR, 0.791; 95% CI, 0.648–0.964; *P* = 0.010) at median follow-up of 37.5 and 36.9 months, respectively.^75^ Triplets based on VD, such as VMP, VCD, or VTD, can also be used at relapse following Rd induction and after fixed duration V-based induction.

The choice between Vd-based regimens should also be made based on efficacy and safety and a patient's comorbidities and preferences. Concerning the challenge of lenalidomide-refractory disease at first relapse, there are a number of options. The lenalidomide dose could be increased if the patient had previously been receiving lenalidomide single agent low dose. Alternatively, the patient could be switched to a PI-based combination, for example, Dara-Vd or Kd. Pomalidomide-dexamethasone is emerging as a new backbone regimen after the first line of therapy, with the addition of daratumumab, or a PI (such as bortezomib), or cyclophosphamide.

Salvage ASCT is not recommended in patients with early relapse, but may be a good option if newer regimens are not available. Time to progression after initial ASCT correlates with OS after first relapse and the duration of PFS after initial ASCT correlates with the duration of PFS and OS after salvage ASCT.^76,77^

*Conclusion*: In relapsed/refractory MM, recommended treatment strategy ideally requires a switch of mechanism of action from that used in the front-line setting, from PI-based to IMiD-based regimens, or vice versa. However, if patients have received V-based or Rd fixed duration with a favorable response, retreatment with PI or IMiD combination could be used. Triplet combination regimens appear to be superior to doublets in terms of prolonging PFS and 2-drug regimens are not recommended for high-risk patients if triplet combinations are available.

### Later relapses

Pomalidomide is increasingly the backbone of treatment at second relapse, although daratumumab monotherapy is being used in some countries (Fig. 1).

At second or subsequent relapse, the ESMO guidelines recommend a pomalidomide plus dexamethasone (PomD)-backbone in combination with cyclophosphamide, ixazomib, bortezomib, daratumumab, or elotuzumab; or daratumumab as monotherapy or in combination; or enrolling the patient into a clinical trial of a novel therapy.^19^

A phase 2 study demonstrated that adding cyclophosphamide to PomD resulted in increased ORR compared with PomD alone among 70 patients refractory to lenalidomide (64.7% vs 38.9%; *P* = 0.0355).^78^ PomD is being investigated in a number of ongoing studies in combination with PIs or mAbs (including CD38) both at early relapse and in more advanced disease. Phase 1 data demonstrated an ORR of 65% with PomVD in patients with prior PI exposure who were refractory to lenalidomide.^79^ The OPTIMISMM trial is studying PomVD in patients with 1 to 3 prior lines of therapy with prior lenalidomide exposure.^80^ After phase 1 study data suggested that the CD38 mAb isatuximab may increase the efficacy of PomD in heavily pretreated patients,^81^ the ICARIA-MM study is investigating isatuximab-PomD versus PomD in patients with ≥2 prior lines of therapy who failed treatment with lenalidomide and a PI alone or in combination.^82^ The phase 1b EQUULEUS study indicated that treatment with daratumumab plus PomD resulted in deep and durable responses in a population of heavily pretreated patients with RRMM.^83^ The APOLLO study is evaluating DaraPomD versus PomD in patients with ≥1 prior line of therapy (IMiD and PI).^84^

The final combined analysis of the GEN501 and SIRIUS studies after approximately 3 years’ follow-up showed that daratumumab monotherapy treatment in heavily pretreated patients resulted in a median OS of 20.5 months and an ORR of 30.4%.^85^

The selection of Pom-D versus daratumumab monotherapy depends on efficacy and safety and the last line of therapy the patient received in order to inform the choice between maintaining immunomodulation (if patient has previously received lenalidomide) or switching to another drug (daratumumab).

Other combinations are being evaluated at later relapse. A phase 1b study of isatuximab-Rd in patients with RRMM who had received ≥2 prior treatment regimens (N = 57) showed an ORR of 56%, and 52% in patients refractory to lenalidomide. At median follow-up of 9 months, median PFS was 8.5 months.^86^

*Conclusion*: Current clinical practice mirrors the recommendations of ESMO, with PomD-backbone combinations and daratumumab monotherapy used at second and subsequent relapse.

## Minimal residual disease

Although minimal residual disease (MRD) is not yet assessed routinely in clinical practice, there is a move in some centers to use MRD to guide treatment decisions. Therefore, there is a need to prospectively and systematically evaluate MRD in future clinical trials of different patient groups, including high-risk individuals. To this end, several ongoing clinical studies include systematic MRD assessments.^87,88^ Future clinical trials should also investigate the optimal timing and frequency of MRD evaluation and the predictive value of MRD negativity at key treatment decision points. MRD assessment should be performed at the time of CR and repeated once a year to confirm sustained MRD negativity. At present, there are no clinical consequences for MRD status other than as a prognostic indicator.

For a patient to be considered truly MRD negative, the consensus is that there should be no detectable disease by next-generation sequencing (NGS) or next-generation flow (NGF) cytometry, and by imaging (PET/CT). This is in agreement with the “imaging negative MRD negative” IMWG consensus criteria.^9^ Ideally, future clinical trials will include both NGS and NGF for MRD assessment, with a sensitivity of 10^−6^, which is more stringent than the current IMWG criteria. Furthermore, reports of MRD negativity should be accompanied by details of the technique and sensitivity used.

The concept of sustained MRD negativity, defined by the IMWG as MRD negativity in the marrow (NGF or NGS, or both) and by imaging, confirmed a minimum of 1 year apart,^9^ will likely be important for guiding treatment duration. However as mentioned above, the frequency of MRD assessment to determine sustained MRD negativity should be prospectively studied. In addition, sustained MRD negativity may be introduced as a stopping rule in clinical trials, requiring the discontinuation of therapy if patients remain MRD negative for several years.

In the future, MRD will likely be considered by regulatory authorities as a surrogate endpoint for drug approval. It is anticipated that MRD will be particularly useful in comparing induction regimens, with assessment performed following induction and ASCT.

*Conclusion*: MRD represents the future treatment goal for patients with MM. Clinical trials are ongoing in order to determine the optimal timing, frequency, and sensitivity of MRD assessments, and to investigate its final value in evaluating treatments and guiding management decisions.

## Future developments

Several novel agents are being investigated in MM. A phase 1b study of the small molecule BCL-2 inhibitor, venetoclax-Vd (N = 66) found an ORR of 67%, with a greater overall response (90%) in patients who were not refractory to bortezomib. Increased ORR was also shown to be related to high *BCL2* expression.^89^ In the phase 2 STORM study of selinexor plus dexamethasone (SelD), ORR was 21% in patients with MM refractory to bortezomib, carfilzomib, lenalidomide, and pomalidomide (quad-refractory), and 20% in those also refractory to daratumumab (penta-refractory).^90^ In addition, the phase 1/2 STOMP study SelVd arm, showed a high ORR (83%) in PI-relapsed or naïve patients with up to 3 prior treatments.^91^ These data provided the rationale for the phase 3 BOSTON study, which is assessing the efficacy and safety of SelVd versus Vd in patients with earlier phase disease and 1 to 3 prior lines of treatment.^92^

Combinations of IMiDs and checkpoint inhibitor mAbs have also been studied. The PD1 inhibitor pembrolizumab has shown activity in combination with PomD, with ORR of 60% and median PFS of 17.4 months at median follow-up of 15.6 months.^93^ A phase 1 trial of pembrolizumab-Rd in heavily pre-treated patients (median 4 prior lines), showed an ORR of 50%; high levels of efficacy were also seen among the 38 lenalidomide-refractory patients.^94^ Two phase 3 studies of pembrolizumab-IMiD combinations in MM were put on hold by the FDA in July 2017 due to increased toxicity.^95^ Ongoing clinical studies of antibody–drug conjugates and CAR-T cell therapies (reviewed by Wolska-Washer et al^96^ and Danhof et al^97^) have also shown promising early results,^98–101^ however data from large, phase 3 studies are needed.

## Conclusion

Survival outcomes in MM have improved markedly over recent years due to the introduction of management strategies including ASCT, PI-, and IMiD-based treatments, and targeted therapy with mAbs. Ever deepening levels of response mean that treatment sequencing will become one of the greatest challenges in the future. Furthermore, the achievement of sustained MRD negativity may prove a significant step in defining cure in MM.

## Acknowledgments

The authors would like to thank Phillippa Curran PhD, Rocket Science Medical Communications Ltd, UK, for medical writing and editorial assistance with manuscript preparation under the guidance of the authors, funded by the European Myeloma Network.
